# Measuring HCC Tumor Size in MRI—The Sequence Matters!

**DOI:** 10.3390/diagnostics11112002

**Published:** 2021-10-28

**Authors:** Marco Armbruster, Markus Guba, Joachim Andrassy, Markus Rentsch, Vincent Schwarze, Johannes Rübenthaler, Thomas Knösel, Jens Ricke, Harald Kramer

**Affiliations:** 1 Department of Radiology, University Hospital, LMU Munich, 81377 Munich, Germany; vincent.schwarze@med.uni-muenchen.de (V.S.); johannes.ruebenthaler@med.uni-muenchen.de (J.R.); jens.ricke@med.uni-muenchen.de (J.R.); harald.kramer@die-radiologie.de (H.K.); 2 Department of General, Visceral, Vascular, and Transplant Surgery, Medical Center of the University of Munich, 81377 Munich, Germany; markus.guba@med.uni-muenchen.de (M.G.); joachim.andrassy@med.uni-muenchen.de (J.A.); 3 Department of General, Visceral and Thoracic Surgery, Klinikum Ingolstadt, 85049 Ingolstadt, Germany; markus.rentsch@klinikum-ingolstadt.de; 4 Institute of Pathology, Ludwig-Maximilians-University, 81377 Munich, Germany; thomas.knoesel@med.uni-muenchen.de

**Keywords:** hepatocellular carcinoma, liver transplantation, MRI, hepatobiliary agents, hepatobiliary phase

## Abstract

Background: The aim of this paper was to assess and compare the accuracy of common magnetic resonance imaging (MRI) pulse sequences in measuring the lesion sizes of hepatocellular carcinomas (HCCs) with respect to the Milan criteria and histopathology as a standard of reference. Methods: We included 45 patients with known HCC who underwent contrast-enhanced MRI of the liver prior to liver transplantation or tumor resection. Tumor size was assessed pathologically for all patients. The MRI protocol contained axial T2-weighted images as well as T1-weighted imaging sequences before and after application of Gd-EOB-DTPA. Tumor diameters, the sharpness of lesions, and the presence of artifacts were evaluated visually on all available MRI sequences. MRI measurements and pathologically assessed tumor dimensions were correlated using Pearson’s correlation coefficient and Bland–Altman plots. The rate of misclassifications following Milan criteria was assessed. Results: The mean absolute error (in cm) of MRI size measurements in comparison to pathology was the smallest for the hepatobiliary phase T1-weighted acquisition (0.71 ± 0.70 cm, r = 0.96) and largest for the T2w turbo-spin-echo (TSE) sequence (0.85 ± 0.78 cm, r = 0.94). The misclassification rate regarding tumor size under the Milan criteria was lowest for the T2w half-Fourier acquisition single-shot turbo spin-echo sequence and the hepatobiliary phase T1w acquisition (each 8.6%). The highest rate of misclassification occurred in the portal venous phase T1w acquisition and T2w TSE sequence (each 14.3%). Conclusions: The hepatobiliary phase T1-weighted acquisition seems to be most accurate among commonly used MRI sequences for measuring HCC tumor size, resulting in low rates of misclassification with respect to the Milan criteria.

## 1. Introduction

Hepatocellular carcinoma (HCC) is the most common primary liver cancer in adults and the third leading cause of cancer-related deaths worldwide, with more than 700,000 newly diagnosed cases per year and an increasing incidence in western countries [1,2]. HCC has an unfavorable prognosis when diagnosed at late stages, as therapeutic approaches are limited [3]. However, if detected early, HCC can be effectively treated and patients can be cured. For the diagnosis of HCC, imaging plays a crucial role, while molecular and histological workup might help in making the correct diagnosis in unclear cases [4]. Possible treatment options with curative intent range from local ablation or surgical resection up to liver transplantation (LT). In advanced stages, therapeutic strategies include transarterial chemoembolization (TACE), selective internal radiation therapy and systemic chemo- and hormonotherapy [5]. 

Both for the diagnosis of HCC and its therapeutic management, in particular the eligibility for liver transplantation, measurements of HCC lesion sizes play a crucial role, and most guidelines define cut-off values on which their recommendations are based. For example, along with other imaging features, the liver imaging reporting and data system (LI-RADS^®^) algorithm depends on the following size cut-off values: <1 cm, 1–2 cm, and >2 cm; to stratify the probability of an unclear lesion with arterial hyperenhancement being HCC [6]. The Organ Procurement and Transplant Network—United Network for Organ Sharing (OPTN/UNOS) provides a standardized approach for selecting patients with HCC for liver transplant [7] and defines 5 cm as a cut-off value for acceptable tumor size for transplant, while smaller lesions may qualify for end-stage liver disease (MELD) exception points [8,9]. Under another important algorithm—the Milan criteria—patients qualify for LT if they bear a single intrahepatic HCC nodule of ≤5 cm, or up to 3 lesions of 1 and 3 cm maximum diameter [10]. Although treatment decisions and prognosis of patients depend heavily on the measured HCC size, little information is available about which imaging sequence and which phase of multiphasic imaging is best suited for performing size measurements of HCCs. While the Milan criteria provide no strict recommendations about the phase or sequence in which to measure [11], OPTN/UNOS [9] recommends the late arterial or portal venous phase. Conversely, the LI-RADS^®^ classification recommends not measuring HCC size in the arterial phase, as diameters may vary due to differences in image acquisition timing and might be misconstrued by perilesional enhancement [6]. 

As even small differences in size measurements of HCCs may lead to a change in patient management—especially regarding suitability for LT—and may consequently affect patient outcome, an exact, non-invasive assessment of HCC size is mandatory. The aim of this study was to evaluate the diagnostic performance of different MRI pulse sequences in measuring HCC lesions, including the hepatobiliary phase T1-weighted acquisition with liver-specific contrast agent, and to evaluate their accuracy using histopathology as the reference standard.

## 2. Materials and Methods

### 2.1. Material and Methods

This retrospective, single-center, HIPAA-compliant study was approved by the institutional review board (#EK-LMU-19-395, 28 January 2014). The study protocol conformed to the ethical guidelines of the 1975 Declaration of Helsinki. No donor organs were obtained from executed prisoners or other institutionalized persons.

### 2.2. Patient Study Group and Settings

We retrospectively measured the tumor size of HCC lesions in 45 patients on different preoperatively acquired MRI pulse sequences and compared these measurements with the histopathologically assessed tumor dimensions after surgery. Out of 114 patients with histologically proven HCC who underwent LT or tumor resection in our tertiary center for liver surgery, 61 patients did not meet the further inclusion criteria of dedicated preoperative liver MRI acquired in our hospital (27 cases) within 90 days prior to surgery (20 cases) with the use of liver-specific contrast agent (14 cases). Five cases with neoadjuvant local therapies, as well as three cases with ambiguousness in the assignment of HCC lesions detected in MRI and the histopathological assessment, were excluded—resulting in a total of 45 patients that constituted the final study group. Patient and clinical characteristics of this study group are summarized in Table 1.

### 2.3. Histopathologic Size Measurement

The size of all liver specimens was measured at the Institute of Pathology at our hospital immediately after resection. Specimens were fixed in buffered formalin but not inflated with embedding medium before gross cutting. A pathologist with more than 20 years of experience (T.K.) aimed to cut the largest tumor section, usually in the center of the tumor mass, and measured the maximal tumor diameter using a straight metal rule. The initial gross measurement was re-evaluated at the time of microscopic evaluation of the tumor and the final size was noted in millimeters.

### 2.4. Magnetic Resonance Imaging (MRI)

MRI was performed on two different 1.5 Tesla scanners (Aera/Avanto; Siemens Healthineers, Erlangen, Germany) using a 32-channel phased-array body and spine coil at the Department of Radiology at our hospital. The MRI examination involved T1-weighted (T1w) and T2-weighted (T2w) imaging sequences. T1w imaging was performed as T1w volumetric interpolated breath-hold examination (VIBE), both pre-contrast and in the arterial, portal venous, venous, and hepatobiliary phase 20 min after contrast injection. T2w imaging included a T2w turbo spin-echo sequence (TSE) and T2w half-Fourier acquisition single-shot turbo spin-echo (HASTE). This protocol is in line with published guidelines from the AASLD/EASL [12]. Detailed MRI sequence parameters are illustrated in Table 2.

### 2.5. Image Data Preparation

First, a radiology consultant with 7 years of experience in abdominal imaging (MA) identified all HCC lesions on all available MRI sequences and evaluated their ambiguousness in the assignment to the histopathological reports. The assignment between MRI and histopathology was considered to be unambiguousness if only one HCC lesion was present both in the MRI and the pathology report or, in case of bi-, tri- or multifocal HCC, if there was a single lesion either in the right or left liver lobe, and if the histopathologic report clearly stated the locations of all measured HCC lesions and their belonging to the right or left liver lobe. The lesion was then marked on the MR images by drawing a region of interest around it. In the case of bifocal HCCs with one lesion in the right and left lobe, the lesion with the largest diameter described in the histopathologic report was chosen and marked.

### 2.6. Lesion Measurements

A radiology consultant with more than 15 years of experience in abdominal imaging (HK), blinded for the histopathologic reports and all available clinical information, measured the largest diameter of each marked HCC lesion on all available transverse MRI pulse sequences., Diameters were measured from outer edge to outer edge of each lesion. In case of encapsulation of lesions, the capsule was included in the measurement. Perilesional signal alterations that clearly represented perfusion alterations were excluded from the measurement. In case of central necrosis, nodule-in-nodule or mosaic architecture of the entire lesion was measured. Besides lesion diameters, the sharpness of lesions was rated on each sequence as 1 = sharply delineated or 2 = presenting with indistinct margins. Each sequence was furthermore visually evaluated for the presence of respiratory motion artifacts as 1 = non-existing/minor or 2 = severe, causing illegible images in which the measurement of lesion diameters could not be performed reliably.

### 2.7. Misclassification Regarding the Milan Criteria

The number of misclassifications of each MRI pulse sequence was assessed separately as incorrectly staged inside of (histopathological outside) or incorrectly staged outside of the Milan criteria (histopathological inside). A lesion was considered to be inside the Milan criteria if (1) in cases with a singular lesion the tumor measured equal to or less than 5 cm, or (2) in case of up to three lesions, if each lesion measured equal or less than 3 cm. A lesion was considered to be outside the Milan criteria if (1) in cases with a singular lesion the tumor measured more than 5 cm, or (2) in case of bi- or trifocal lesions, if one of the lesions measured more than 3 cm. To avoid bias due to an unequal distribution of datasets, all examinations were excluded from this analysis if one or more MRI pulse sequence was missing (e.g., due to technical failures or patient interruption of the examination).

### 2.8. Statistical Analysis

All data were analyzed using commercially available software (Stata IC 13.1; StataCorp LP, College Station, TX, USA; GraphPad Prism version 7.04 for Windows, GraphPad Software, La Jolla, CA, USA). To confirm whether continuous variables were normally distributed, the Kolmogorov–Smirnov test was applied for diameters in each of the measured MRI sequences (T1w and T2w pre-contrast, T1w post-contrast). The size on each MRI sequence was correlated with histopathological measurements using Pearson correlation coefficients. Correlation coefficients (r) were considered to be non-existing for r < 0.20, weak 0.21–0.40, moderate 0.41–0.60, strong 0.61–0.80 and almost perfect > 0.80. Paired t tests were used to compare sequences in terms of the absolute error between pathology as a standard of reference and MRI measurements. Bland–Altman plots were used to assess agreement between pathologic and MRI measurements for each sequence. All *p* values were two-sided and considered statistically significant at *p* < 0.05.

## 3. Results

The mean size of the 45 included HCCs based on histopathologic assessment was 7.2 ± 3.4 cm (range: 1.5 cm to 14.0 cm).

### 3.1. HCC Visibility and Image Artifacts

The percentage of HCC lesions that were visible was highest for the arterial, venous, and hepatobiliary phase (all 100%; Table 3). The lowest sensitivity for visualizing HCC was achieved with the T2w HASTE sequence (90%). Lesions presented most often with indistinct circumscription on T1-weighted acquisition in the arterial phase (52%), while they were most often sharply circumscribed in the hepatobiliary phase (85%). Respiratory motion artifacts occurred most often on the T2w TSE sequence (10%), while they occurred in none of T1-weighted pre-contrast and hepatobiliary phase images.

### 3.2. Pearson Correlation and Absolute Error

**Figure 1:**
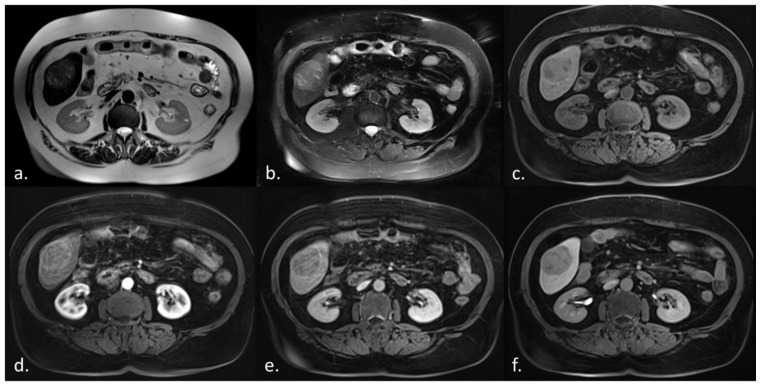
HCC size measurements vary between different MRI pulse sequences. Figure 1 shows an HCC in the right liver lobe measuring 4.9 cm in the T2w half-Fourier-acquired single-shot turbo spin echo sequence (**a**), 5.2 cm in the T2w turbo spin-echo sequence (**b**), 4.9 cm in the T1w volumetric interpolated breath-hold examination sequence (**c**–**f**) pre-contrast (**c**), 5.3 cm in the arterial phase (**d**), 5.2 cm in the venous phase (**e**), and 4.8 cm in the hepatobiliary phase (**f**). Histopathologic assessment revealed a tumor diameter of 4.5 cm.

There was an almost perfect correlation between all MRI sequences and histopathology (all r > 0.94, *p* < 0.001, Table 4). The hepatobiliary phase T1-weighted acquisition showed the highest correlation coefficient (r = 0.96), while the other phases showed slightly lower correlation coefficients (r = 0.94–0.95). The absolute error between MRI and the histopathologic assessment was lowest for the hepatobiliary phase (0.71 ± 0.70 cm), and highest for T2w TSE (0.85 ± 0.78 cm). An example of the variance of size measurements among different MRI pulse sequences is shown in Figure 1.

### 3.3. Misclassification Regarding the Milan and OPTN/UNOS Criteria

The most important point for transplantation eligibility is a tumor size within the Milan criteria (≤3 cm and ≤5 cm, respectively (11)). A total of 5 out the 35 patients were misclassified in at least one sequence (Table 5). Within this group, the most misclassifications occurred for the T2w TSE sequence and in the portal venous phase T1w acquisition (five cases each), followed by the pre-contrast, arterial phase and venous phase T1w acquisition (four cases each). The lowest rate of misclassifications was found for the T2w HASTE sequence and in the hepatobiliary phase T1w acquisition (three cases each).

### 3.4. Bland–Altman Assessment

**Figure 2:**
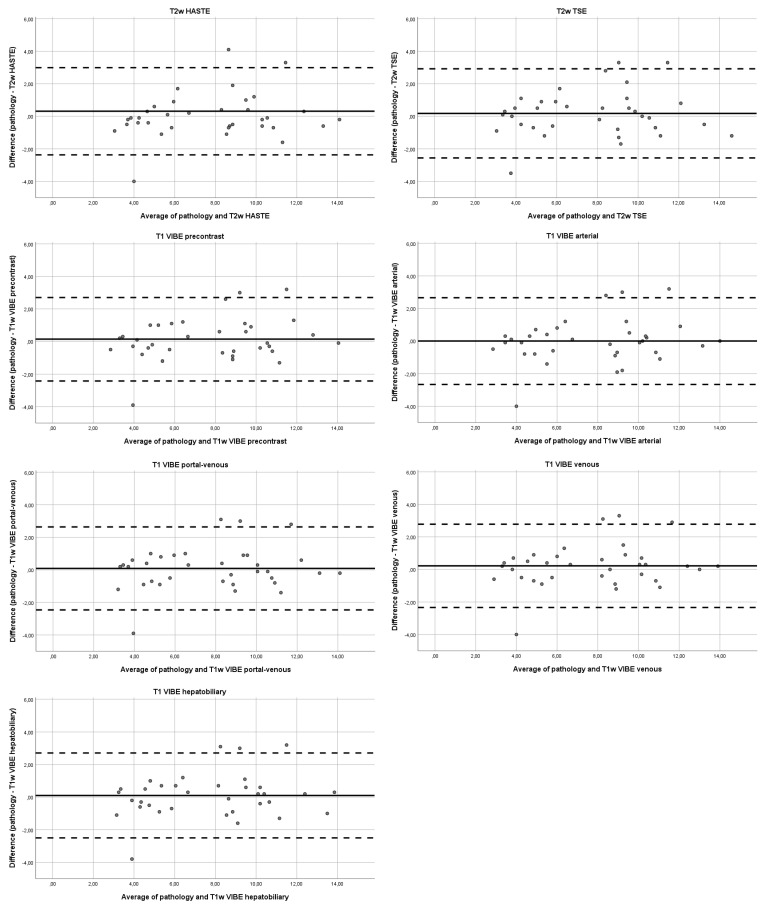
Bland–Altman plots of the average and difference of pathology versus the different MRI sequences.

As seen in Figure 2, and summarized in Table 6, the T2w HASTE sequence had a systematic mean under-estimation of HCC size (−0.13 cm), while the arterial phase T1-weighted acquisition and T2w TSE sequence showed a systematic over-estimation of HCC tumor size (+0.41 cm and +0.20 cm, respectively). The lowest mean difference was found for the portal venous phase (+0.10 cm), pre-contrast (+0.11 cm) and hepatobiliary phase (+0.12 cm) with T1-weighted acquisition. The 95% limits of agreement relative to histopathologic assessment were widest for the arterial phase (−2.37 to +2.45) and narrowest for the hepatobiliary phase (−1.84 to +2.09).

## 4. Discussion

There is high demand for precise and valid radiologic size measurements of HCC, as most therapy regimens and in particular the eligibility for liver transplantation highly depend on the size of intrahepatic HCC lesions. While the OPTN/UNOS criteria define 5 cm as the cut-off value as the acceptable tumor size for transplant, the Milan criteria qualifies patients for LT only if there is a single intrahepatic HCC nodule ≤ 5 cm or up to 3 lesions between 1 cm and 3 cm [8,9,10].

Although MRI is considered to be the radiologic reference standard to detect and diagnose HCC, no conclusive recommendation has been announced by the leading societies as to which MRI sequence should be used when performing size measurements [12,13,14,15,16]. While the OPTN/UNOS guidelines recommend measuring the lesion in the late arterial phase, the LI-RADS^®^ guidelines propose using the sequence in which the lesion is most clearly visualized whilst avoiding the late arterial phase, and the AASLD guideline provides no further specification regarding the MRI phase [6,9,12,16].

Our study showed that HCC size measurements in MRI differ significantly between commonly used sequences and that this has an impact upon the rate of misclassifications of tumor diameter in consideration to the current guidelines for transplant eligibility. The T2w TSE and portal venous phase T1-weighted acquisition classified 14.3% of the lesions in an incorrect Milan category, whereas the T2w HASTE and hepatobiliary phase T1-weighted acquisition reduced this error to 10.8%. However, the T2w HASTE also had the lowest detection rate of HCCs in our study, and is therefore not suitable if the lesion is not visible in this sequence. On the other hand, the hepatobiliary phase T1-weighted acquisition offered the highest sensitivity for HCCs and additionally showed the best correlation with histopathological size measurements and a low rate of Milan misclassifications. Furthermore, in this phase, the lowest mean absolute error and the best image quality were achieved.

In our study cohort, the arterial phase T1-weighted acquisition and T2w TSE sequence systematically overestimated HCC size (on average +0.41 cm and +0.20 cm, respectively). One possible explanation for this trend is hyperemic and edematous changes in the tissue surrounding the HCC lesions, which can appear isointense to the tumor on the arterial phase and the T2w TSE sequence. On the other hand, the T2w HASTE sequence slightly underestimated the HCC size (−0.13 cm). However, both trends were not statistically significant—possibly due to the low power of the current study, with only 45 patients. Further studies with a larger study cohort are necessary to confirm these findings. Furthermore, differences in size measurements between histopathology and all analyzed MRI pulse sequences might also be explained by volume alterations of tissue samples due to the histological fixation process.

Seuss et al. have previously shown that the sizes of lesions vary between different MRI sequences and different phases in comparison to histopathologic results, with the highest accuracy in the portal venous phase [17]. This contradicts the findings of our study, which could not identify any advantages of the portal venous phase regarding the accuracy of size measurements. A possible explanation may be the use of different contrast agents. While Seuss et al. evaluated MRI phases with an extracellular, non-liver specific contrast agent (Gd-BT-DO3A), in our study, a liver specific contrast agent (Gd-EOB-DTPA) was used. Currently, the major guidelines for the diagnosis of HCC still recommend the use of extracellular contrast agents as the primary method; however, they also see the need for further studies to assess diagnostic performance with the use of hepatobiliary agents [5,9,18]. Therefore, we decided to use a hepatobiliary agent that has a high hepatobiliary uptake and biliary excretion, which results in a faster and stronger hepatobiliary signal [19,20,21,22]. Another advantage of the use of hepatobiliary agents in comparison to extracellular agents is the higher detection rate of HCCs, as they may lack arterial hyperenhancement—especially in cases of well-differentiated HCCs [18,22].

Severe liver diseases with impaired liver function such as cirrhosis or cholestasis can affect the validity of the hepatobiliary phase due to a reduced uptake of the contrast agent and altered signal intensity differences [23,24]. Focal liver lesions such as HCCs are usually characterized by a reduced uptake of hepatobiliary contrast agent. Consequently, there are also studies showing an impairment of MRI with hepatobiliary agents with increasing cirrhosis [18,25]. This may have affected the results in our study, as in four out of five cases with misclassifications regarding the Milan criteria, patients suffered from cirrhosis. A further limitation of this study is the use of non-isotropic sequences which do not allow the generation of multiplanar reconstructions. As all size measurements were performed on truly axial or coronal planes, non-spherical lesions might not have been measured along their largest diameter but rather along their short axis. However, this reflects the real-life setting of most liver MRI protocols and affected all sequences in a similar manner. Therefore, this systemic error might affect our results on the absolute differences between MRI and histopathologic size measurements, but not on the comparison between the different MRI sequences and different MRI phases.

## 5. Conclusions

Our data suggest that commonly used MRI pulse sequences differ in their accuracy in measuring HCC tumor size, with potential misclassifications following current guidelines for transplant eligibility. Consequently, our findings highlight the need of a consensus on how we measure HCC size to prevent unequal treatment of patients between different physicians, departments and hospitals.

## Figures and Tables

**Table 1:** Characteristics of the study group (*n* = 45). Mean age ± SD was 63 ± 14 years (range 20–81).

Characteristics	N
Sex	
Male	36
Female	9
Amount of HCC lesions	
Unifocal	36
Bifocal	4
Trifocal	3
Multifocal	2
Risk factors	
Cirrhosis	16
Early stage fibrosis	9
HCV	8
HBV	5
Hemochromatosis	1
Hepatic porphyria	1
Surgical procedure	
Right hemihepatectomy	11
Left hemihepatectomy	1
Extended right hemihepatectomy	1
Segment resection	13
Atypical resection	3
LT	16

HCV: Hepatitis C Virus; HBV: Hepatitis B Virus; LT: Liver transplantation.

**Table 2:** Parameters of MRI pulse sequences.

	Slice Thickness (mm)	Matrix	TE (ms)	TR (ms)	FA (°)	BW (Hz)
T2 TSE	6	270 × 320	105	2730	180	220
T2 HASTE	6	320 × 384	78	800	180	390
T1 VIBE *	3	230 × 256	1.35	3.44	15	455

TE: Time to echo; TR: Repetition time; FA: Flip angle; BW: Bandwith; TSE: Turbo spin echo; HASTE: Half-Fourier acquisition single-shot turbo-spin-echo; VIBE: Volumetric interpolated breath-hold examination. * Sequence parameters were constant both for pre-contrast and contrast-enhanced series (arterial, portal-venous, venous and delayed phase).

**Table 3:** Percentage of HCC visible on each available MRI sequence.

Sequence	Percentage of HCCs Visible	Percentage of Lesions with Fuzzy Circumscription	Percentage of Artifacts with Each Sequence
T2w HASTE	37/41 (90%)	16/41 (43%)	1/41 (2%)
T2w TSE	40/42 (95%)	15/42 (38%)	4/42 (10%)
T1w precontrast	42/44 (95%)	9/44 (21%)	0/44 (0%)
Arterial phase	44/44 (100%)	23/44 (52%)	2/44 (5%)
Portal venous phase	43/45 (96%)	19/45 (44%)	3/45 (7%)
Venous phase	44/44 (100%)	21/45 (47%)	1/44 (2%)
Hepatobiliary phase	41/41 (100%)	6/41 (15%)	0/41 (0%)

HCC: Hepatocellular carcinoma; HASTE: Half-Fourier acquisition single-shot turbo spin-echo; TSE: Turbo spin echo. Not all sequences were acquired in every patient due to technical failures and patient interruption of the examination. All HCCs were visible in the arterial, venous, and hepatobiliary phase. Regarding image quality, the hepatobiliary and T1w precontrast phase provided best image quality with less artifacts.

**Table 4:** Absolute error and Pearson correlation coefficient (r) between MRI and histopathologic measurements.

Sequence	Mean ± SD (cm)	95% CI (cm)	Range (cm)	r
T2w HASTE	0.83 ± 0.81	0.57–1.09	0.1–3.3	0.94
T2w TSE	0.87 ± 0.82	0.62–1.13	0.0–3.7	0.94
T1w precontrast	0.79 ± 0.77	0.56–1.02	0.0–3.2	0.95
Arterial phase	0.81 ± 0.92	0.55–1.08	0.0–3.8	0.94
Portal venous phase	0.82 ± 0.85	0.57–1.07	0.0–3.5	0.94
Venous phase	0.78 ± 0.84	0.53–1.03	0.0–3.5	0.95
Hepatobiliary phase	0.75 ± 0.75	0.52–0.97	0.0–3.3	0.96

CI: Confidence interval, HASTE: Half-Fourier acquisition single-shot turbo spin-echo; TSE: Turbo spin echo. All sequences achieved a high correlation coefficient between MRI and histopathologic measurements, with the hepatobiliary phase being the most accurate, showing the highest correlation coefficient and lowest absolute mean error.

**Table 5:** Misclassification regarding the Milan criteria (*n* = 35).

Category	T2w HASTE	T2w TSE	T1w VIBE Pre-Contrast	T1w VIBE Arterial	T1w VIBE pv	T1w VIBE Venous	T1w VIBE HPB
Pathologically inside Milan Criteria							
Unifocal ≤ 5 cm (8)	1	2	1	2	2	2	1
Up to three ≤ 3 cm (1)	1	1	1	1	1	1	1
Pathologically outside of Milan Criteria							
At least one > 5 cm (25)	1	2	2	1	2	1	1
Bi-/Trifocal > 3 cm (1)	0	0	0	0	0	0	0
Total	8.6% (3)	14.3% (5)	11.4% (4)	11.4% (4)	14.3% (5)	11.4% (4)	8.6% (3)

HASTE: Half-Fourier acquisition single-shot turbo spin-echo; TSE: Turbo spin echo; pv: Portal-venous; VIBE: Volumetric interpolated breath-hold sequence; HPB: Hepatobiliary. With respect to Milan criteria, in the T2w TSE sequence and T1w VIBE portal venous phase 5 lesions (14.3%) were categorized in the wrong Milan category. The lowest number of misclassifications was found in the T2w HASTE sequence and T1w VIBE hepatobiliary phase (3 lesions; 8.6%). Only datasets were included in this analysis in which all MRI pulse sequences were available (*n* = 35).

**Table 6:** Bland–Altman assessment of different MRI sequences vs. pathology.

Sequence	Mean Difference (cm)	95% Limits of Agreement (cm)	Range (cm)
	95% CI		
T2w HASTE	−0.13	−2.45 to 2.19	2.30 to 14.20
	−0.52 to 0.27		
T2w TSE	+0.20	−2.06 to 2.45	1.20 to 15.20
	−0.17 to 0.56		
T1w precontrast	+0.11	−2.02 to 2.25	1.50 to 14.10
	−0.22 to 0.45		
Arterial phase	+0.41	−2.37 to 2.45	1.6 to 14.0
	−0.33 to 0.41		
Portal venous phase	+0.10	−2.16 to 2.36	1.50 to 14.20
	−0.25 to 0.46		
Venous phase	+0.22	−1.92 to 2.35	1.60 to 13.80
	−0.11 to 0.55		
Hepatobiliary phase	+0.12	−1.84 to 2.09	1.50 to 14.00
	−0.19 to 0.44		

HASTE: Half-Fourier acquisition single-shot turbo spin-echo; TSE: Turbo spin echo. In the Bland–Altman analysis the hepatobiliary phase provided the narrowst 95% limits of agreement (−1.84 to 2.09 cm) with respect to histopathologic assessment as a reference standard. While the arterial phase, on average, overestimated tumour size (+0.41 cm), T2w HASTE tended to slighly underestimate HCC size (−0.13 cm); however, these trends are not statistically significant.

## Data Availability

The data presented in this study are available on request from marco.armbruster@med.uni-muenchen.de. The data are not publicly available due to privacy and ethical restrictions.
